# Regional anaesthesia in patients on antithrombotic drugs – a joint ESAIC/ESRA guideline: Endorsement by the Scandinavian Society of Anaesthesiology and Intensive Care Medicine

**DOI:** 10.1111/aas.14093

**Published:** 2022-05-30

**Authors:** Morten Hylander Møller, Martin Ingi Sigurðsson, Klaus T. Olkkola, Marius Rehn, Arvi Yli‐Hankala, Michelle S. Chew

**Affiliations:** ^1^ Department of Intensive Care Copenhagen University Hospital Rigshospitalet Copenhagen Denmark; ^2^ Division of Anesthesia and Intensive Care Medicine Landspitali ‐The National University Hospital of Iceland Reykjavik Iceland; ^3^ Faculty of Medicine University of Iceland Reykjavik Iceland; ^4^ Department of Anaesthesiology, Intensive Care and Pain Medicine Helsinki University Hospital, University of Helsinki Helsinki Finland; ^5^ Division of Prehospital Services, Air Ambulance Department Oslo University Hospital Oslo Norway; ^6^ The Norwegian Air Ambulance Foundation Oslo Norway; ^7^ Faculty of Health Sciences University of Stavanger Stavanger Norway; ^8^ Department of Anaesthesia Tampere University Hospital Tampere Finland; ^9^ Faculty of Medicine and Health Technology Tampere University Tampere Finland; ^10^ Department of Anaesthesia and Intensive Care, Biomedical and Clinical Sciences Linköping University Linköping Sweden

**Keywords:** AGREE II, antithrombotic drugs, bleeding, clinical practice guideline, regional anaesthesia

## Abstract

The Clinical Practice Committee of the Scandinavian Society of Anaesthesiology and Intensive Care Medicine endorses the clinical practice guideline *Regional anaesthesia in patients on antithrombotic drugs – a joint ESAIC/ESRA guideline.* This clinical practice guideline serves as a useful decision aid for Nordic anaesthesiologists providing regional anaesthesia to adult patients on antithrombotic drugs.

## 1 BACKGROUND

A rare but feared complication to regional anaesthesia is bleeding.^1^ The risk of bleeding is increased in patients on antithrombotic agents, and many patients undergoing regional anaesthesia today are elderly with co‐existing diseases and on antithrombotic drugs.^2^

The clinical practice guideline *Regional anaesthesia in patients on antithrombotic drugs – a joint ESAIC/ESRA guideline* provides evidence‐based recommendations for providing regional anaesthesia in adult patients on antithrombotic drugs.^3^

## 2 METHODS

The Clinical practice committee (CPC) of the Scandinavian Society of Anaesthesiology and Intensive Care Medicine (SSAI) decided to assess the clinical practice guideline *Regional anaesthesia in patients on antithrombotic drugs – a joint ESAIC/ESRA guideline* ^3^ for possible endorsement. The Appraisal of Guidelines for REsearch and Evaluation (AGREE) II tool^4^ was used. Details on the endorsement process are available elsewhere.^5^

## 3 RESULTS

**FIGURE 1:**
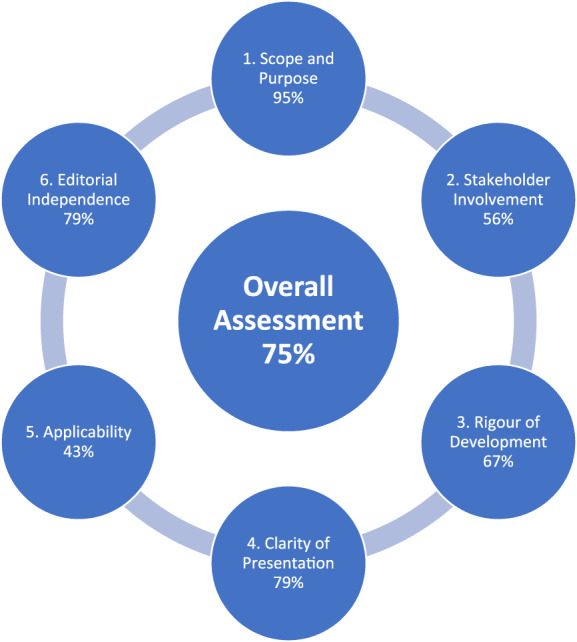
Summary of the Appraisal of Guidelines for REsearch and Evaluation (AGREE) II assessment^4^

All six SSAI CPC members completed the appraisal. The individual domain totals were: Scope and Purpose 95%; Stakeholder Involvement 56%; Rigour of Development 67%; Clarity of Presentation 79%; Applicability 43%; Editorial Independence 79%; Overall Assessment 75% (Figure 1).

The breakdown of the individual appraisers (de‐identified) is available in Supporting Information Material S1.

## 4 DISCUSSION

Agreement between the SSAI CPC appraisers was acceptable and the overall assessment of the guideline was good. There were issues related to stakeholder involvement and applicability, which were only covered briefly. Furthermore, the timing of post‐procedural dosing of antithrombotic agents was not covered in detail, and readers need to refer elsewhere for guidance on this.^6^, ^7^ This likely has to do with the fact that timing of post‐procedural dosing of antithrombotic agents depends on the specific type and dose of drug used, renal function and whether it is administered in combination with antiplatelet agents or not.

The guideline can be used in daily clinical practice in the Nordic countries without major adaptation or modification, noting the limitations in applicability above.

The clinical practice guideline *Regional anaesthesia in patients on antithrombotic drugs – a joint ESAIC/ESRA guideline* ^3^ serves as a useful decision aid for Nordic anaesthesiologists providing regional anaesthesia to adult patients on antithrombotic drugs.

## 5 CONCLUSION

The SSAI CPC endorses the clinical practice guideline *Regional anaesthesia in patients on antithrombotic drugs – a joint ESAIC/ESRA guideline*.^3^

## AUTHOR CONTRIBUTION

All authors drafted, revised and approved the manuscript.

## Supporting information


**Appendix S1** Supporting Information
