# Supplementary material for: Regional anaesthesia in patients on antithrombotic drugs – a joint ESAIC/ESRA guideline: Endorsement by the Scandinavian Society of Anaesthesiology and Intensive Care Medicine
Source: Acta Anaesthesiol Scand. 2022 May 30;66(7):887–9. doi: 10.1111/aas.14093 (PMC9546463; doi:10.1111/aas.14093)
Supplement: Supplementary file 1 — Appendix S1 Supporting Information [file AAS-66-887-s001.pdf]

# A critical group appraisal of: Regional anaesthesia in patients on antithrombotic drugs using the AGREE II Instrument

Created with the AGREE II Online Guideline Appraisal Tool.

No endorsement of the content of this document by the AGREE Research Trust should be implied.

Co-ordinator: Morten Hylander Møller

Date: 11 January 2022

Email: mortenhylander@gmail.com

URL of this appraisal: http://www.agreetrust.org/group-appraisal/15741

Guideline URL:

| Domain 1 | Domain 2 | Domain 3 | Domain 4 | Domain 5 | Domain 6 | OA 1 | OA 2 |
|---|---|---|---|---|---|---|---|
| 95% | 56% | 67% | 79% | 43% | 79% | 75% | Yes - 4, Yes with modifications - 2, No - 0 |

| *Domain 1. Scope and Purpose* | | | | | | |
|---|---|---|---|---|---|---|
| | Appraiser 1 | Appraiser 3 | Appraiser 7 | Appraiser 2 | Appraiser 5 | Appraiser 4 |
| Item 1 | 7 | 7 | 7 | 6 | 7 | 7 |
| Item 2 | 7 | 6 | 7 | 6 | 7 | 7 |
| Item 3 | 7 | 6 | 7 | 6 | 7 | 7 |
| | | | | | | |
| *Domain 2. Stakeholder Involvement* | | | | | | |
| | Appraiser 1 | Appraiser 3 | Appraiser 7 | Appraiser 2 | Appraiser 5 | Appraiser 4 |
| Item 4 | 3 | 5 | 7 | 6 | 4 | 6 |
| Item 5 | 3 | 4 | 2 | 1 | 2 | 2 |
| Item 6 | 3 | 6 | 7 | 6 | 5 | 6 |
| | | | | | | |
| *Domain 3. Rigour of Development* | | | | | | |
| | Appraiser 1 | Appraiser 3 | Appraiser 7 | Appraiser 2 | Appraiser 5 | Appraiser 4 |
| Item 7 | 7 | 6 | 7 | 6 | 7 | 7 |
| Item 8 | 7 | 6 | 7 | 6 | 7 | 6 |
| Item 9 | 4 | 7 | 5 | 6 | 2 | 3 |
| Item 10 | 6 | 6 | 7 | 6 | 6 | 7 |
| Item 11 | 6 | 6 | 7 | 6 | 6 | 5 |
| Item 12 | 4 | 6 | 7 | 6 | 2 | 6 |
| Item 13 | 6 | 4 | 1 | 1 | 1 | 6 |
| Item 14 | 1 | 2 | 1 | 2 | 1 | 3 |
| | | | | | | |
| *Domain 4. Clarity of Presentation* | | | | | | |
| | Appraiser 1 | Appraiser 3 | Appraiser 7 | Appraiser 2 | Appraiser 5 | Appraiser 4 |
| Item 15 | 4 | 6 | 7 | 6 | 2 | 6 |
| Item 16 | 5 | 6 | 7 | 7 | 6 | 5 |
| Item 17 | 7 | 5 | 7 | 6 | 7 | 4 |
| | | | | | | |
| *Domain 5. Applicability* | | | | | | |
| | Appraiser 1 | Appraiser 3 | Appraiser 7 | Appraiser 2 | Appraiser 5 | Appraiser 4 |

| | | | | | | |
|---|---|---|---|---|---|---|
| Item 18 | 1 | 5 | 7 | 4 | 4 | 4 |
| Item 19 | 1 | 5 | 7 | 5 | 2 | 2 |
| Item 20 | 1 | 4 | 2 | 5 | 1 | 5 |
| Item 21 | 1 | 4 | 7 | 6 | 1 | 2 |
| | | | | | | |
| *Domain 6. Editorial Independence* | | | | | | |
| | Appraiser 1 | Appraiser 3 | Appraiser 7 | Appraiser 2 | Appraiser 5 | Appraiser 4 |
| Item 22 | 1 | 5 | 7 | 6 | 4 | 7 |
| Item 23 | 7 | 6 | 7 | 6 | 7 | 6 |
| | | | | | | |
| *Overall Assessment* | | | | | | |
| | Appraiser 1 | Appraiser 3 | Appraiser 7 | Appraiser 2 | Appraiser 5 | Appraiser 4 |
| OA1 | 5 | 5 | 6 | 6 | 5 | 6 |

Created online at www.agreetrust.org 11 January 2022
